# *Oreoglanis hponkanensis*, a new sisorid catfish from north Myanmar (Actinopterygii, Sisoridae)

**DOI:** 10.3897/zookeys.646.11049

**Published:** 2017-01-17

**Authors:** Xiao-Yong Chen, Tao Qin, Zhi-Ying Chen

**Affiliations:** 1 Southeast Asia Biodiversity Research Institute, Chinese Academy of Sciences, Yezin, Nay Pyi Taw 05282, Myanmar; 2 State Key Laboratory of Genetic Resources and Evolution, Kunming Institute of Zoology, Chinese Academy of Sciences, Kunming, Yunnan 650223, China

**Keywords:** Hponkanrazi, Irrawaddy, Myanmar, Siluriformes

## Abstract

During a survey of the Mali Hka River drainage in Hponkanrazi Wildlife Sanctuary in December 2015, a new species was collected and is described herein as *Oreoglanis hponkanensis*. It is a member of the *Oreoglanis siamensis* species group and can be distinguished from its congeners in having a unique combination of the following characters: lower lip with median notch and posterior margin entire, caudal fin emarginate, nasal barbel reaching about half the distance to eye, tip of maxillary barbel rounded, posterior margin of maxillary barbel entire, absence of pale elliptical patches on sides of body below adipose fin, absence of patch on base of first dorsal fin ray, caudal fin brown with two round, bright orange patches in middle, branched dorsal fin rays 5, branched anal fin rays 2, vertebrae 40, pectoral fin surpassing pelvic fin origin, pelvic fin length 21–26% SL, caudal peduncle length 25–33% SL, caudal peduncle depth 3–5% SL, adipose fin base length 34–39% SL, and dorsal to adipose distance 12–16% SL.

## Introduction

The Sisoridae is the largest family of Asian catfish, with more than 200 species and 22 genera (Ferraris 2007; Ng 2015). Members are found along the entire southern arc of the Asian continent and comprise a significant portion of the hill-stream fauna in southern and eastern Asia (Ng and Jiang 2015). Recent morphological (Ng 2015) and molecular research (Ng and Jiang 2015) reconstructed the monophyly of Sisoridae and divided it into Sisorinae and Glyptosterninae subfamilies.The Sisorinae includes 12 genera in three tribes (Bagariini, Erethistini and Sisorini). The Glyptosterninae includes 10 genera in one tribe (Glyptosternini).The Glyptosterninae is well-supported as a monophyletic group with 15 synapomorphies, within which *Oreoglanis* is monophyletic and considered to be a sister group of *Pseudoexostoma* and *Exostoma*, with five synapomorphies (Ng 2015), and a sister group of *Creteuchiloglanis* and *Pseudoexostoma* (Ng and Jiang 2015).

The genus *Oreoglanis* was established by Smith (1933) for glyptosternine catfish characterized with a continuous postlabial groove in the lower jaw and an unusual dentition of pointed teeth in the upper jaw and posterior part of the lower jaw and truncate-spatulate teeth in the anterior part of the lower jaw (Ng and Kottelat 1999). There are currently 22 valid species of *Oreoglanis* (Ng and Rainboth 2001; Ng and Freyhof 2001; Ng 2004; Kong et al. 2007; Vidthayanon et al. 2009; Linthoingambi and Vishwanath 2011; Sinha and Tamang 2015). Among them, only *Oreoglanis macropterus* and *Oreoglanis insignis* have been recorded from the Irrawaddy River drainage of Myanmar and China. During a survey of the Mali Hka River drainage in Hponkanrazi Wildlife Sanctuary in December 2015, we collected specimens of *Oreoglanis*, which we identified as a new species and describe herein as *Oreoglanis hponkanensis*.

## Materials and methods

Measurements were made point to point with dial calipers and recorded to 0.2 mm. Counts and measurements were made on the left side of the specimens when possible. Subunits of the head were measured as proportions of head length (HL). Head length and body parts were measured as proportions of standard length (SL). Counts and measurements followed Ng and Kottelat (1999). Vertebral counts followed Roberts (1994). Images of tooth bands, maxillary barbels, and genital papillae were taken with an Olympus SZ61 and ToupCam microscope digital camera. Radiographs were obtained to count vertebrae using a digital Cabinet X-ray System (Kubtec Xpert 80). The examined specimens are deposited at the Kunming Institute of Zoology (KIZ), Chinese Academy of Sciences (CAS), Kunming, China, and the Southeast Asia Biodiversity Research Institute (SEABRI), Chinese Academy of Sciences, Nay Pyi Taw, Myanmar.

## Results

### Oreoglanis hponkanensis sp. n.

Taxon classificationAnimaliaSiluriformesSisoridae

http://zoobank.org/A539FAAD-34D9-4370-ABCA-D6048BC54CA6

Figure 1

#### Holotype.

KIZ2015006376 (CXY20150125), 102.14 mm SL; Myanmar: Kachin State, Hponkanrazi Wildlife Sanctuary, Zeyar Stream near Zeyar Dan Village, 27°34.2'N, 97°06.05'E; XY. Chen, T. Qin and SS. Shu, 14 Dec. 2015.

#### Paratypes.

KIZ2015006375 (CXY20150124), KIZ2015006377 (CXY20150126), 2 ex., 78.88–99.26 mm SL; data as for holotype. SEABRI-CXY20150143, 1 ex., 110.68 mm SL; Myanmar: Kachin State, Hponkanrazi Wildlife Sanctuary, Ponyin Stream near Zeyar Dan Village, 27°33.86'N, 97°05.42'E; XY. Chen, T. Qin and SS. Shu, 14 Dec. 2015. SEABRI-CXY20150104, SEABRI-CXY20150106, KIZ2015006378, 3 ex., 70.6–120.64 mm SL; Myanmar: Kachin State, Hponkanrazi Wildlife Sanctuary, Zeyar Stream near Zeyar Dan Village, 27°34.2'N, 97°06.05'E; XY. Chen, T. Qin and SS. Shu, 9 Dec. 2015. SEABRI-CXY20150078, 1 ex., 88.78 mm SL; Kachin State, Hponkanrazi Wildlife Sanctuary, Monlar Stream near Warsar Dan Village, 27°29.82'N, 97°11.34'E; XY. Chen, T. Qin and SS. Shu, 7 Dec. 2015.

#### Diagnosis.

*Oreoglanis hponkanensis* is a member of the *Oreoglanis siamensis* species group, and can be distinguished from its congeners in having a unique combination of the following characters: lower lip with median notch and posterior margin entire, caudal fin emarginate, nasal barbel reaching about half the distance to eye, tip of maxillary barbel rounded, posterior margin of maxillary barbel entire, absence of pale elliptical patches on sides of body below adipose fin, absence of patch on base of first dorsal fin ray, caudal fin brown with two round, bright orange patches in middle, branched dorsal fin rays 5, branched anal fin rays 2, vertebrae 40, pectoral fin surpassing pelvic fin origin, pelvic fin length 21–26% SL, caudal peduncle length 25–33% SL, caudal peduncle depth 3–5% SL, adipose fin base length 34–39% SL, and dorsal to adipose distance 12–16% SL.

#### Description.

Morphometric data are listed in Table 1. Head and body moderately broad and very strongly depressed. Mouth and gape inferior, with broad and thin papillate lips. Lower lip with median notch, posterior margin entire. Postlabial groove on lower jaw present and uninterrupted. Jaw teeth pointed, in a large broad band with small median indentation and rounded ends on both sides of upper jaw. Teeth on lower jaw present in two, well-separated patches of roughly triangular shape and of two kinds: anterior teeth truncate-spatulate, inner face curved; posterior teeth pointed like those of upper jaw (Figure 2). Eyes small, dorsolaterally situated and subcutaneous. Gill openings extending to middle of pectoral fin base. Maxillary barbels flattened, with surrounding flap of skin and rounded tip; ventral surface with numerous plicae; posterior margin of maxillary barbel entire (Figure 3D). Nasal barbel short, reaching about half the distance to eye.

**Figure 1.**
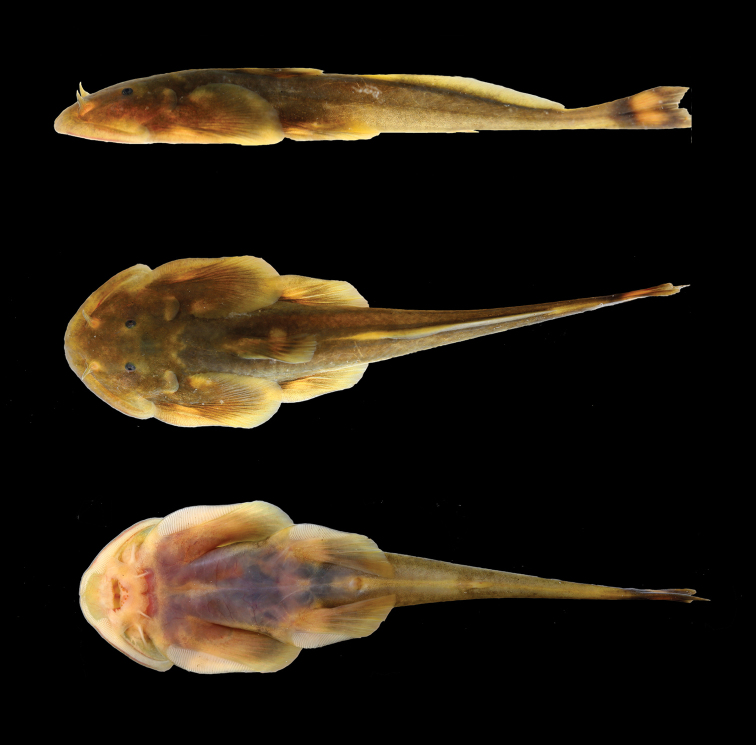
*Oreoglanis hponkanensis*, SEABRI CXY20150104, paratype, male, 70.6 mm SL.

**Figure 2.**
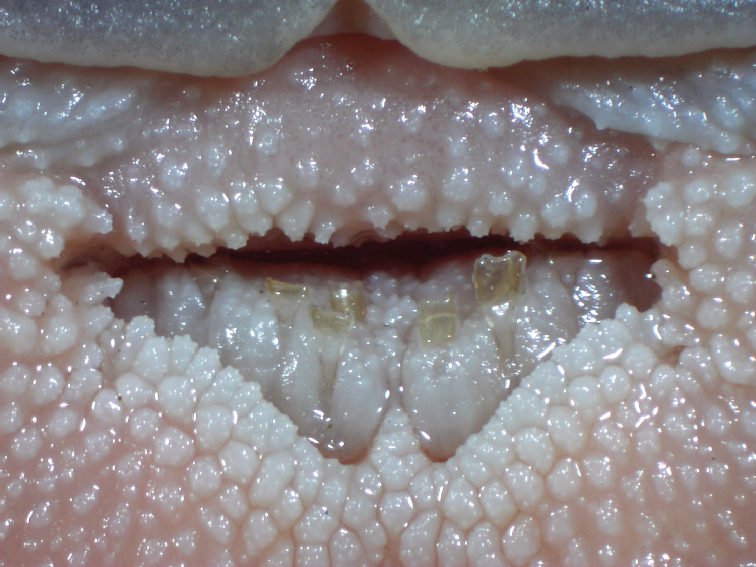
Tooth band of *Oreoglanis hponkanensis*, SEABRI CXY20150106, paratype, male, 120.64 mm SL.

**Figure 3.**
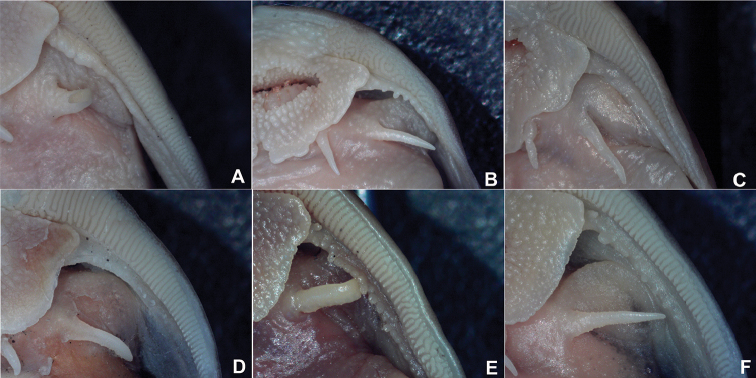
Comparison of posterior margin of maxillary barbel of *Oreoglanis* species. **A** *Oreoglanis jingdongensis*, KIZ 200104003, holotype, 109.1 mm SL **B** *Oreoglanis immaculatus*, KIZ 200261015, holotype, 54.2 mm SL **C** *Oreoglanis insignis*, KIZ 9810191, holotype, 66.7 mm SL **D** *Oreoglanis hponkanensis*, KIZ CXY20150126, paratype, 78.88 mm SL **E** *Oreoglanis macropterus*, KIZ 2004000834, 79.6 mm SL **F** *Oreoglanis setiger*, KIZ 2016000859, 90.0 mm SL.

**Table 1.** Morphometric data of *Oreoglanis hponkanensis* sp. n. (n = 8).

Catalog number	Holotype	Range	Mean	SD
Total length (mm)	114.3	80.1–135.0	–	–
Standard length (mm)	102.1	70.6–120.6	–	–
**Percentage of standard length**				
Head length	18.5	16.5–22.9	20.0	2.53
Head width	18.3	17.4–23.2	19.8	2.28
Head depth	8.4	8.1–10.3	9.0	0.91
Predorsal length	25.6	24.2–30.9	28.3	2.61
Prepectoral length	12.5	11.2–16.4	13.4	1.92
Prepelvic length	32.3	29.2–34.6	32.7	2.28
Preanal length	61.3	61.3–69.3	66.2	2.93
Body depth at anus	9.6	9.3–12.0	10.3	1.05
Caudal peduncle length	29.9	25.4–33.2	29.7	2.99
Caudal peduncle depth	3.6	3.1–4.8	3.8	0.58
Dorsal to adipose distance	15.8	11.5–16.0	14.4	1.79
Post adipose length	11.2	8.4–11.9	10.4	1.27
Dorsal fin base length	9.0	7.8–10.6	9.3	1.11
Adipose fin base length	36.8	36.3–39.2	37.5	1.19
Pectoral fin length	25.0	23.4–29.0	26.3	2.28
Pelvic fin length	21.0	20.5–25.9	23.1	2.11
Anal fin base length	3.1	3.1–5.5	3.8	1.00
**Percentage of head length**				
Head width	1.0	0.9–1.1	1.0	0.04
Head depth	0.5	0.4–0.5	0.5	0.03
Snout length	0.7	0.6–0.7	0.6	0.03
Interorbital width	0.3	0.3–0.3	0.3	0.02
Eye diameter	0.1	0.1–0.1	0.1	0.01
Nasal barbel length	0.2	0.2–0.2	0.2	0.03
Maxillary barbel length	1.0	0.8–1.1	0.9	0.08
Outer mandibular barbel length	0.2	0.2–0.2	0.2	0.01
Inner mandibular barbel length	0.2	0.1–0.2	0.2	0.03

Dorsal fin without spine and with i, 5 (7) rays. Adipose fin with long base. Anal fin with i, 2 (7) rays. Caudal fin emarginate, with 6/6 (7) rays. Pelvic fin greatly enlarged, with convex distal margin and i, 5 (7) rays; first ray flattened, with numerous plicae on ventral surface; tip of pelvic fin surpassing anus, and anus at midpoint between posterior end of pelvic fin base and tip of pelvic fin. Pectoral fin greatly enlarged, without spine and with i, 16 (4) or i, 17 (3) rays; first ray flattened, with numerous plicae on ventral surface. Tip of pectoral fin reaching beyond pelvic fin origin; Vertebrae 25+15=40 (3), or 26+14=40 (1).

**Figure 4.**
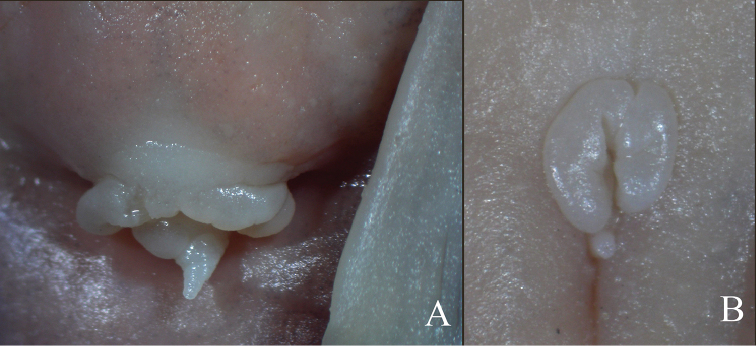
Ventral view of anus and external genital papilla of *Oreoglanis hponkanensis*. **A** SEABRI CXY20150106, paratype, male, 120.64 mm SL **B** SEABRI CXY20150078, paratype, female, 88.78 mm SL.

Males with small genital papilla located immediately posterior to anus (Figure 4A). Females with two flaps of skin on both sides of anus, and small genital papilla located in longitudinal groove immediately posterior to anus (Figure 4B).

**Color.** In life: brown on dorsal and lateral surfaces of head and body, light yellow on ventral region. Dorsal surfaces of head and body with series of small, light yellow patches: two ovoid patches on occipital region, elliptical patches on anterior and posterior bases of adipose fin. Ovoid patch on base of first dorsal fin ray absent, and elliptical patch on lateral surface of body below middle part of adipose fin base absent. Dorsal fin brown, dorsal surfaces of pectoral and pelvic fins brown, anal fin and ventral surfaces of pectoral and pelvic fins light yellow. Adipose fin light yellow. Caudal fin brown with two round, bright orange patches in middle. Pectoral fin base occasionally with round yellow patch on inner and outer anterior sides, respectively. Dorsal surface of barbels brown, ventral surface light yellow.

#### Distribution.

**Figure 5.**
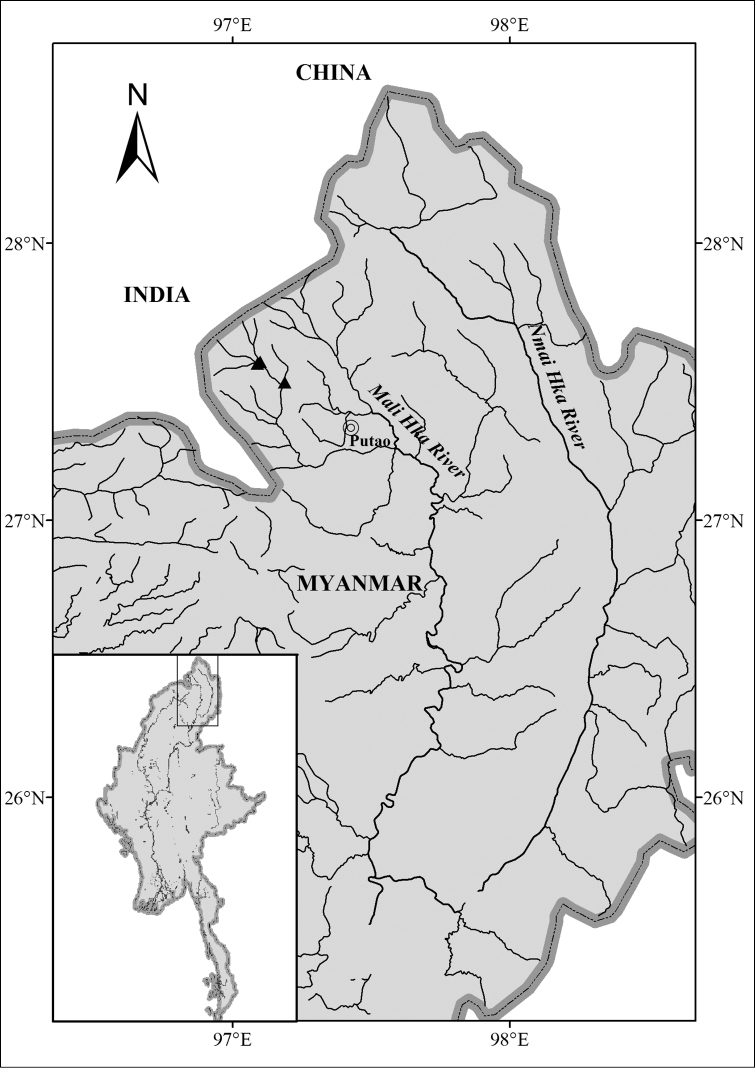
Distribution map of *Oreoglanis hponkanensis*.

Known from high mountain streams of Mali Hka River drainage (upper Irrawaddy River drainage) in Hponkanrazi Wildlife Sanctuary, Kachin State, north Myanmar (Figure 5).

#### Habitat.

**Figure 6.**
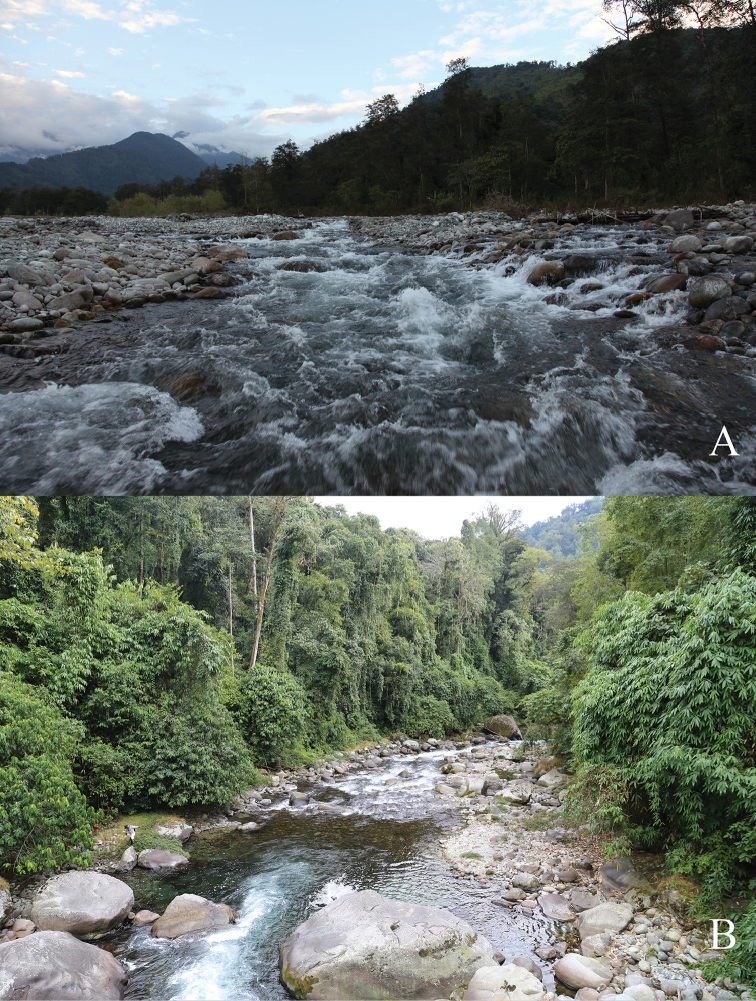
Habitat of *Oreoglanis hponkanensis*. **A** Zeyar Stream, Mali Hka drainage, Hponkanrazi Wildlife Sanctuary, north Myanmar **B** Monlar Stream, Mali Hka drainage, Hponkanrazi Wildlife Sanctuary, north Myanmar.

Fast flowing mountain streams with stone, cobble, and sand beds (Figure 6A, B). Other associated fish species recorded from the type locality include: Cyprinidae: *Danio aequipinnatus*, *Barilius barnoides*, *Tor qiaojiensis*, *Neolissochilus* sp., *Garra salweenica*, *Garra bispinosa*, *Placocheilus dulongensis*, *Schizothorax meridionalis*; Nemacheilidae: *Paracanthocobitis adelaideae*, *Schistura malaisei*; Siluridae: *Pterocryptis berdmorei*; Amblycipitidae: *Amblyceps murraystuarti*; Sisoridae: *Exostoma vinciguerrae*; Channidae: *Channa burmanica*.

#### Etymology.

From Hponkanrazi Wildlife Sanctuary, adjectival.

## Discussion

*Oreoglanis hponkanensis* is a member of the *Oreoglanis siamensis* species group (*sensu* Ng and Rainboth 2001) based on the presence of a lower lip with a median notch. There are 13 species in this group, including *Oreoglanis hponkanensis*. *Oreoglanis hponkanensis* can be distinguished from its congeners in the *Oreoglanis siamensis* species group by branched anal fin rays 2 vs. 3–6.

*Oreoglanis hponkanensis* shares with *Oreoglanis immaculatus*, *Oreoglanis insignis*, *Oreoglanis jingdongensis*, *Oreoglanis laciniosus*, *Oreoglanis majusculus*, *Oreoglanis macropterus*, *Oreoglanis pangenensis*, *Oreoglanis setiger*, and *Oreoglanis suraswadii* in having the tip of the maxillary barbel rounded and the pectoral fin reaching or surpassing pelvic fin origin (vs. tip of maxillary barbel pointed and pectoral fin not reaching pelvic fin origin in *Oreoglanis siamensis*, *Oreoglanis sudarai*, and *Oreoglanis heteropogon*).

*Oreoglanis hponkanensis* is further distinguished from *Oreoglanis siamensis* in having the posterior margin of lower lip entire (vs. with small laciniate projections), the posterior margin of the maxillary barbel entire (vs. lobulated), longer pelvic fin (surpassing vs. not reaching anus; length 21–26% SL vs. 15–16), absence (vs. presence) of pale patches on the sides of the body below the adipose fin, fewer branched dorsal fin rays (5 vs. 6), longer and more slender caudal peduncle (length 25–33% SL vs. 17–23; depth 3–5% SL vs. 5–6), shorter nasal barbel (16–25% HL vs. 28–37), and larger interorbital distance (28–34% HL vs. 23–27).

*Oreoglanis hponkanensis* can be further distinguished from *Oreoglanis sudarai* in having the posterior margin of lower lip entire (vs. with lobulate projections), longer pelvic fin (greatly surpassing anus vs. slightly surpassing), absence (vs. presence) of pale patches on sides of body below adipose fin absent, longer caudal peduncle (length 25–33% SL vs. 17–23), shorter prepelvic length (29–35% SL vs. 37–43), shorter anal fin base length (3–6% SL vs. 6–9), longer pelvic fin length (21–26% SL vs. 13–17), and shorter nasal barbel (16–25% HL vs. 27–41).

*Oreoglanis hponkanensis* can be distinguished from *Oreoglanis heteropogon* in having the nasal barbel reaching midway between its base and anterior orbital (vs. reaching anterior margin of orbit), the posterior margin of the maxillary barbel entire (vs. with crenulate projections), more branched dorsal fin rays (5 vs. 6), more vertebrae (40 vs. 38), shorter predorsal length (24–31% SL vs. 35), shorter prepectoral length (11–16% vs. 19), shorter prepelvic length (29–35% SL vs. 42), shorter preanal length (61–69% SL vs. 75), shorter dorsal to adipose distance (12–16% SL vs. 20), longer caudal peduncle (length 25–33% SL vs. 18), shorter post-adipose distance (8–12% SL vs. 13), longer adipose fin base (34–39% SL vs. 29), longer pelvic fin (21–26% SL vs. 13), shorter anal fin base (3–6% SL vs. 7), larger interorbital distance (28–34% HL vs. 22), and shorter nasal barbel (16–25% HL vs 33).

*Oreoglanis hponkanensis* differs from *Oreoglanis jingdongensis* and *Oreoglanis suraswadii* in having the caudal fin emarginate (vs. lunate). It can be further distinguished from *Oreoglanis jingdongensis* in having the posterior margin of the maxillary barbel entire (vs. with crenulate projections, Figure 3A), more slender and narrower caudal peduncle (length 25–33% SL vs. 20–26, depth 3–5% SL vs. 5–8), pelvic fin surpassing anus for a longer distance (vs. just surpassing), less blunt snout, and fewer vertebrae (40 vs. 42–43). It can be further differentiated from *Oreoglanis suraswadii* in having the pelvic fin greatly surpassing anus (vs. just surpassing), more vertebrae (40 vs. 36–38), absence (vs. presence) of pale patches on sides of body below adipose fin, longer caudal peduncle (25–33% SL vs. 19–25), shorter prepelvic length (29–35% SL vs. 36–40), and shorter dorsal fin base (8–11% SL vs. 11–14).

*Oreoglanis hponkanensis* can be distinguished from *Oreoglanis laciniosus* in having the posterior margin of the lower lip entire (vs. with lobulate projections), absence (vs. presence) of pale patches on sides of body below adipose fin, shorter predorsal length (24–31% SL vs. 35–37), shorter prepelvic length (29–35% SL vs. 38–42), and longer adipose fin base (34–39% SL vs. 32–33).

*Oreoglanis hponkanensis* shares a similar color pattern with *Oreoglanis immaculatus*, but differs in having the posterior margin of the lower lip entire (vs. with lobulate projections), the posterior margin of the maxillary barbel entire (vs. with laciniate projections, Figure 3B), more vertebrae (40 vs. 37–38), longer pelvic fin (21–26% SL vs. 18–21), pelvic fin far surpassing anus (vs. just surpassing), much slenderer caudal peduncle (length 25–33% SL vs. 17–21, depth 3–5% SL vs. 5–7), longer adipose fin base (34–39% SL vs. 26–33), and shorter dorsal to adipose distance (12–16% SL vs. 16–23).

*Oreoglanis hponkanensis* differs from *Oreoglanis macropterus* in having the posterior margin of the maxillary barbel entire (vs. with lobulate projections, Figure 3E), absence (vs. presence) of pale patches on sides of body below adipose fin, fewer branched dorsal fin rays (5 vs. 6), fewer caudal fin rays (6/6 vs. 7/8, 8/7, or 8/8), shorter maxillary barbel (surpassing posterior edge of eye vs. closer to gill opening), much slenderer caudal peduncle (length 25–33% SL vs. 19–22 and depth 3–5% SL vs. 8–9), and shorter dorsal to adipose distance (12–16% SL vs. 18–19).

*Oreoglanis hponkanensis* can be distinguished from *Oreoglanis majusculus* in having the posterior margin of the maxillary barbel entire (vs. with villiform projections), absence (vs. presence) of patches on sides of body below adipose fin, fewer branched pectoral fin rays (16–17 vs. 20), fewer caudal fin rays (6/6 vs. 7/8), and much slenderer caudal peduncle (with length 25–33% SL vs. 18–21 and depth 3–5% SL vs. 6).

*Oreoglanis hponkanensis* differs from *Oreoglanis pangenensis* in having the posterior margin of the maxillary barbel entire (vs. with lobulate and laciniate projections), fewer branched dorsal fin rays (5 vs. 6), fewer caudal fin rays (6/6 vs. 7/8), absence (vs. presence) of pale patches on sides of body below adipose fin, slenderer caudal peduncle (with length 25–33% SL vs. 23 and depth 3–5% SL vs. 5), shorter head (17–23% SL vs. 26), shorter predorsal length (24–31% SL vs. 33), shorter preanal length (61–69% SL vs. 75), shorter prepelvic length (29–35% SL vs. 36), longer adipose fin base (34–39% SL vs. 31), and shorter dorsal to adipose distance (12–16% SL vs. 21).

*Oreoglanis hponkanensis* can be differentiated from *Oreoglanis setiger* in having the posterior margin of the lower lip entire (vs. with laciniate projections), the posterior margin of the maxillary barbel entire (vs. with laciniate projections, Figure 3F), fewer branched dorsal fin rays (5 vs. 6), anus much closer to snout tip than to caudal fin base (vs. vice versa), absence (vs. presence) of pale patches on sides of body below adipose fin, more vertebrae (40 vs. 36), much slenderer caudal peduncle (with length 25–33% SL vs. 15–16), shorter predorsal length (24–31% SL vs. 32–37), shorter prepelvic length (29–35% SL vs. 37–39), and larger eye diameter (8–12% HL vs. 7–8).

*Oreoglanis hponkanensis* can be distinguished from *Oreoglanis insignis* in having the distance between anal fin origin and caudal fin base almost equal to distance between pelvic and anal fin origins (vs. almost equal to distance between posterior end of pelvic fin base and anal fin origin), anus much closer to snout tip than caudal fin base (vs. anus at midpoint between snout tip and caudal fin base), absence (vs. presence) of pale patches on sides of body below adipose fin, black (vs. yellow) tip of caudal fin, fewer branched dorsal fin rays (5 vs. 6), fewer caudal fin rays (6/6 vs. 8/7), more vertebrae (40 vs. 36–39), shorter predorsal length (24–31% SL vs. 31–35), shorter prepelvic length (29–35% SL vs. 36–40), and longer adipose fin base (34–39% SL vs. 29–34).

Within species of the *Oreoglanis siamensis* group, *Oreoglanis siamensis* and *Oreoglanis sudarai* only occur in the Chao Phraya River drainage, *Oreoglanis suraswadii*, *Oreoglanis setiger*, and *Oreoglanis jingdongensis* only occur in the Mekong River drainage, *Oreoglanis heteropogon*, *Oreoglanis laciniosus*, and *Oreoglanis immaculatus* are found only in the Salween River drainage, *Oreoglanis majusculus* and *Oreoglanis pangenensis* only occur in the Brahmaputra River drainage, *Oreoglanis macropterus* occurs in the Salween and Irrawaddy river drainages, and *Oreoglanis insignis* and *Oreoglanis hponkanensis* are only found in the Irrawaddy River drainage. Ng and Rainboth (2001) erroneously stated that *Oreoglanis insignis* was distributed in the “upper Irrawaddy and Salween (Nu Jiang) river drainages in northern Myanmar and southwestern China” based on specimens from René Malaise’s 1934 collection “Qushi, Baoshan, Yunnan, China from the Kambawti area, Kachin state, Myanmar and Tengchong area, Yunnan, China”. Kambawti, Tengchong, and Qushi (a Tengchong Township) are located in the Irrawaddy drainage. Thus, *Oreoglanis insignis* should be confined to the Irrawaddy drainage, as clarified by Chen (2013).

### Key to *Oreoglanis siamensis* group

**Table:** 

1	Tip of maxillary barbel pointed; tip of pectoral fin not reaching pelvic fin origin	**2**
–	Tip of maxillary barbel rounded; tip of pectoral fin reaching or surpassing pelvic fin origin	**4**
2	Nasal barbel reaching midway between its base and anterior orbital margin	***Oreoglanis siamensis***
–	Nasal barbel reaching anterior orbital margin	**3**
3	Pectoral fin not reaching pelvic fin origin	***Oreoglanis heteropogon***
–	Pectoral fin reaching pelvic fin origin	***Oreoglanis sudarai***
4	Caudal fin lunate	**5**
–	Caudal fin emarginate	**6**
5	Upper and lower caudal fin first principal rays of approximately equal length	***Oreoglanis suraswadii***
–	Lower first principal ray of caudal fin much longer than upper	***Oreoglanis jingdongensis***
6	Posterior margin of lower lip with lobulate projections	**7**
–	Posterior margin of lower lip entire	**10**
7	Posterior margin of maxillary barbel entire	***Oreoglanis laciniosus***
–	Posterior margin of maxillary barbel with lobulate or laciniate projections	**8**
8	Posterior margin of maxillary barbel with lobulate projections; yellow patch below adipose fin absent	***Oreoglanis immaculatus***
–	Posterior margin of maxillary barbel with laciniate projections; yellow patch below adipose fin present	***Oreoglanis setiger***
10	Posterior margin of maxillary barbel entire	**11**
–	Posterior margin of maxillary barbel with projections	**12**
11	Distance between anal fin origin and caudal fin base almost equal to distance between pelvic and anal fin origins; anus much closer to snout tip than caudal fin base; patches on sides of body below adipose fin absent	***Oreoglanis hponkanensis* sp. n.**
–	Distance between anal fin origin and caudal fin base almost equal to distance between posterior end of pelvic fin base and anal fin origin; anus at midpoint between snout tip and caudal fin base; patch on sides of body below adipose fin present	***Oreoglanis insignis***
12	Posterior margin of maxillary barbel villiform; ovoid patch on base of first dorsal fin ray absent	***Oreoglanis majusculus***
–	Posterior margin of maxillary barbel lobulate; ovoid patch on base of first dorsal fin ray present	**13**
13	Posterior margin of maxillary barbel lobulate; caudal peduncle depth 8–9% SL	***Oreoglanis macropterus***
–	Posterior margin of maxillary barbel with lobulate and laciniate projections; caudal peduncle depth 5% SL	***Oreoglanis pangenensis***

## Comparative material

*Oreoglanis immaculatus*. Holotype: KIZ 200261015, 54.2 mm SL, Paratypes: KIZ200261010, 012, 014, 016, 4 ex., 57.4–63.9 mm SL, Nanjing River (a tributary of the upper Salween), Yongde County, Yunnan, China; KIZ 794762, KIZ794763, 2 ex., 52.0–54.3 mm SL, Nangun River (a tributary of the upper Salween), Cangyuan County, Yunnan, China.

*Oreoglanis insignis*. Holotype: KIZ 9810191, 66.7 mm SL, Longchuanjiang River (a tributary of the upper Irrawaddy), Tengchong County, Yunnan, China. KIZ 2006010198-217, 220-229, 30 ex., 41.–85.3 mm SL, upper Binglang River (upper Daying River, a tributary of the Irrawaddy), Tengchong County, Yunnan, China.

*Oreoglanis jingdongensis*. Holotype: KIZ 200104003, 109.1 mm SL, Paratypes: KIZ 200104001-002, 004-008, 7 ex., 87.1–115.2 mm SL, upper Mengpian River (a tributary of the upper Mekong), Jingdong County, Yunnan, China.

*Oreoglanis macropterus*. KIZ 2004000749-754, 825-840, 22 ex., 43.8–85.1 mm SL, a tributary of upper Dulong River (a tributary of the upper Irrawaddy), Gongshan County, Yunnan, China.

*Oreoglanis setiger*. KIZ 2016000859, 868, 870, 874, 883, 5 ex., 74.7–95.0 mm SL, Nanbi River (a tributary of the upper Mekong), Mengsa Township, Gengma County, Yunnan, China.

## Supplementary Material

XML Treatment for Oreoglanis hponkanensis
